# Book Reviews

**Published:** 1889-10

**Authors:** 


### Book Reviews.

---

Cyclopedia of the Diseases of Children, Medical and Surgical. By John M. Keating, M. D., containing articles from American, Canadian and British authors. J. B. Lippincott Co., 1889. Volume 1 of this work is now before the profession. This volume of nearly one thousand pages, well illustrated and clearly printed, is meeting with much favor. The ability of Dr. Keating is too well known for me to say that his able efforts are eminently successful.

The Anatomy of Children, both general and local, with splendid illustrations by Dr. George McClellan, is very complete. Each organ is shown, and its relation to surrounding organs; also external landmarks are given, showing difference in position of parts in infantile and adult life. Following the anatomy, we have the physiology ably handled, showing when the various glands begin to act, and what effect they produce. Special attention is also given to increase (during health) of weight with each month and various measurements, all of which are important.

Great care is given to physical diagnosis and its methods as applicable to infants.

Many diseases of the fœtus are pointed out and a number of valuable illustrations adorn this part of the work.

Dr. Penrose, under the head of Care of the Child at Birth, goes thoroughly into the method of washing, dressing and handling the child. In tying the cord, Dr. Penrose says: "The ligature should be applied about the breadth of three fingers from the child's body; that is, far enough to allow for re-tying, should any accident render this necessary, and not so near as to cause the true skin of the child caught in the tie." Whether the doctor means to leave the cord the length of three finger breadths, if no re-tying is necessary, he doesn't state. It appears to me that three finger breadths is too long, and that one and a half inches would be better, as we want to promote dessication.

Taking up abnormal conditions, he first speaks of DEBILITY IN THE NEW BORN. When the child is pale or blue, Dr. Penrose doesn't believe in exhausting these feeble, fainting children, for they are often too feeble to withstand a vigorous bath. If a bath is used, he recommends it to be 110 degrees F. to 112 degrees F., and in some cases recommends whisky to be added. In CONJESTIVE ASPHYXIA when the brain and medulla are congested, Dr. Penrose allows from one to three tablespoons of blood to flow from cord before ligating.

Under the head of INFANT FEEDING AND WEANING, we have an able paper by Dr. T. M. Rotch. He thinks the mother's milk is better for the infant than any substitute that can be prepared, unless grave changes occur, due to perverted function of the mother. If it is necessary to take the child from the mother's breast for a short time, he recommends a wet nurse.

He also calls special attention to too frequent or too seldom nursing. If child nurses too often it renders the milk too concentrated; on the other hand if too long intervals between nursing the milk is not nutritious.

Dr. Rotch thinks that several chemical analyses should be demanded of the mother's milk before we resort to a wet nurse or to prepared food as a substitute. If it is necessary to take the child from its mother's milk, and it is impossible to procure a suitable wet nurse, he recommends the milk of cows to be used, as it approaches nearest to human milk, and can be had most anywhere. Dr. Rotch gives the constituents of most of the patent baby foods, showing the merits and disadvantages of each, directing how each should be used. He also strongly advocates sterilization of any mixtures that may be given a child. Under this heading he presents attractive illustrations, and his preparation of cow's milk and his cream mixture is very instructive. In speaking of weaning, Dr. Rotch says that date will usually settle itself, and he is much opposed to sudden weaning. He prefers to get a food that will beyond a doubt agree with the child, then gradually withdraw the mother's milk; he also opposes weaning children during the summer months, much preferring the winter, and if for any reason we wean the child before the eruption of the molars, its food should be milk.

The chapter on Dentition, by Dr. John Dorning, is especially instructive, giving the development of the teeth, and date their appearance. Dr. Dorning is opposed to laying so many ills of the teething child solely to dentition or the reflexes therefrom; he rather regards them as concomitant with dentition. He regards intestinal disturbances due to improper feeding, bad hygiene or excessive heat, rather than to dentition. Dr. Dorning is opposed to rubbing the child's gums with thimbles and other hard substances, as they do no good, but often inflame the gums. He doesn't believe in lancing the gums to relieve pain or tension, as he believes the act of eruption in healthy children to be painless.

PART II. This portion is devoted to fever and miasmatic diseases, and will be read with much interest, as it is ably written and each article gives the latest researches, going thoroughly into bacteria and their causative relation to the various diseases discussed. This portion contains an article on cerebro-spinal fever, also one on diphtheria, by Dr. J. Lewis Smith. He goes thoroughly into his subjects. He does not believe that there is an active microbe the cause of cerebro-spinal fever, but says if there be a microbe, as has been suggested, it is of a very low order. He rather regards poor sanitary surroundings as one of the strongest factors in its production.

He also believes that excessive mental and physical exertions may predispose. Dr. Smith, in his treatment of cerebro-spinal fever, strongly recommends proportionately large doses of bromide of potash, also ergót, to be given in the early stages. Dr. Smith thinks that a great number of cases of diphtheria are contracted from sewer gas or from sewer emanations. In speaking of the particular microbe that produces diphtheria, all the supposed discovered germs are mentioned and described, but Dr. Smith rather believes that no particular microbe causes the toxic effects seen in the disease, but rather that they (bacteria) produce poisonous ptomaines which are absorbed and poison the system.

He mentions Dr. Prudden's streptococus, which caused considerable comment a few months since.

Dr. Smith gives three forms of the lesion in the throat, one in which the mucous membrane becomes necrotic, becomes incorporated and forms a part of the pseudo-membrane; one in which the pseudo-membrane covers the mucous membrane, but is separate from it; and another in which no membrane occurs, catarrhal. Virchow and his followers restricted the use of the term diphtheritic to that form of inflammation in which mucous membrane undergoing necrosis forms part of the pseudo-membrane, the term croupus to the now adherent form ; hence the confusion of the terms diphtheria and croup.

Dr. Smith does not believe that every case of diphtheria is followed by paralysis. He thinks that one out of every three or four cases will have some paralysis during malignant epidemics, but in ordinary epidemics one in eight or ten.

The papers on eruptive fever, also malaria, are very thorough.

It is impossible for us to bring before the profession all the points of interest so ably set forth in the work, but we take great pleasure in recommending it. B. W. B.

---

INEBRIETY, ITS ETIOLOGY, PATHOLOGY, TREATMENT AND JURISPRUDENCE. By Norman Kerr, M. D., F. L. S., London, 1889.

We opened this book with anticipations of pleasure, thinking to find from an author whose name had such a pyramid of titles and honors following it, a scientific treatise on a subject of interest. The book is disappointing. It is neither scientific, learned, wise, practical nor reliable. In it is found not the utterance of a man seeking truth, nor a *résumé* of cases accurately reported from which we can deduce conclusions, nor a description of new or trustworthy methods of treatment, but only the specious verbiage of a special pleader, ending with a covert advertisement of the particular institution to which the author is consulting physician.

The writer of this book starts by assuming a dogma, and upon this builds his structure, bending all facts, illustrations, statements and advice to fit his assumed premise. He claims that "inebriety is a disease," that it belongs to the group of "diseases of the nervous system," and "its nearest ally is insanity." Upon what argument are these statements based? Upon none, forsooth, not one. He apologetically says that "many inebriates are more sinned against than sinning, who are so constituted that to drink in what is called 'moderation' is beyond their power." We grant that, but it is an idiosyncrasy, not a disease. No man is obliged to drink, even in moderation. If so constituted as Dr. Kerr states, why drink at all.

When we read the chapters on symptoms and pathology we find nothing more than the manifestations of alcoholism. There is no distinct train of morbid phenomena, either clinically or *post mortem*, to distinguish "inebriety" so called from acute or chronic alcoholic poisoning. It is plain that this new named disease is not "a disease of the nervous system." It is a poisoning which secondarily breaks down the nervous system. As well say measles is a disease of the eye or ear because it affects these structures secondarily, or scarlet fever, or diphtheria a disease of the kidneys because we find albuminuria.

We might continue this criticism by pointing out the many inconsistencies in the author's statements. We might inquire how he can reconcile his statements about tobacco with his theory of alcohol. We might ask why he does not give reasons for the faith that is in him, and not try to foist his foundling ideas, named and christened out of wedlock, upon a confiding public and profession.

Dr. Kerr's statements are pernicious. Any statements are untrustworthy which have no facts to sustain them. But when we find that the outcome of his book is to advise compulsory retention in a retreat of which the Dalrymple Home (to which Dr. Kerr is physician) is the best, the reasons for this treatise are not difficult to discover.

"Inebriety" is a failure as a scientific treatise; it is useless as a guide to practice, since its teachings can only be followed when one has compulsory control of the patient; and as a book of reference it contains nothing trustworthy but in connection with England.

T. H. H.

---

QUESTIONS AND ANSWERS ON THE ESSENTIALS OF OBSTETRICS PREPARED ESPECIALLY FOR STUDENTS OF MEDICINE. By Wliliam Easterly Ashton, M. D., Philadelphia, W. B. Saunders, 1888.

Another quiz compend by a new aspirant for fame and fortune. It does very well—especially for students of Jefferson College, who will find Prof. Parvin's ideas very correctly given. These short books are always pernicious but this is no worse than the rest of them. They only serve to cram the student with a mass of indigestible matter.

